# The novel multiple sclerosis susceptibility gene *ATXN1* regulates B cell receptor signaling in B-1a cells

**DOI:** 10.1186/s13041-020-00715-0

**Published:** 2021-01-21

**Authors:** Qin Ma, Alessandro Didonna

**Affiliations:** Department of Neurology, Weill Institute for Neurosciences, University of California, 675 Nelson Rising Lane, San Francisco, CA 94158 USA

**Keywords:** Multiple sclerosis, Ataxin-1, B-1a cells, Autoimmunity

## Abstract

Multiple sclerosis (MS) is an autoimmune demyelinating disease of the central nervous system (CNS) caused by complex gene-environment interactions. *ATXN1* maps to 6p22.3, within the 233 loci associated with an increased risk of developing MS. Toxic gain-of-function mutations in *ATXN1* cause the neurodegenerative disorder spinocerebellar ataxia type 1 (SCA1). Conversely, *ATXN1* loss-of-function is involved in Alzheimer’s disease (AD) and tumorigenesis. We have recently shown that *ATXN1* exerts a protective immunomodulatory activity in the MS model experimental autoimmune encephalomyelitis (EAE). Specifically, we demonstrated that mice lacking *Atxn1* experience aggravated EAE due to aberrant B cell functions. *Atxn1*-null mice exhibit increased B cell proliferation with the concomitant expansion of specific B cell subsets including B-1a cells. This population of B cells is responsible for the production of natural immunoglobulins and has been associated with the etiology of multiple autoimmune diseases. To understand the role played by *Atxn1* in these cells, we performed comprehensive transcriptomic profiling of *Atxn1*-null B-1a cells before and after stimulation with an encephalitogenic antigen. Importantly, we show that in this sub-population *Atxn1* regulates immunoglobulin gene transcription and signaling through the B cell receptor (BCR).

## Background

B cell activation plays a central pathogenic role in the chronic, central nervous system autoimmune disease multiple sclerosis (MS) through several, non-mutually exclusive mechanisms that include the production of antibodies and other neurotoxic products, and antigen presentation with consequent propagation of neuroinflammation [1]. The administration of B cell depleting antibodies to people with MS has made a major impact on the management of the disease, definitely confirming their role in the expression, perhaps also initiation of the autoimmune response [2]. Decoding the genetic regulation of B cell function is therefore necessary to further our understanding of disease pathogenesis and advance next generation therapies. We have recently shown that ataxin-1—a polyglutamine protein implicated in the etiology of spinocerebellar ataxia type 1 (SCA1), Alzheimer’s disease (AD), and various types of cancer [3–5]—exerts a B cell-mediated protective effect on the experimental autoimmune encephalomyelitis (EAE) model [6]. This effect is mediated by regulating the extracellular signal-regulated kinase (ERK) and signal transducer and activator of transcription (STAT) pathways. Noteworthy, *ATXN1* is the most plausible MS associated gene in the 6p22.3 disease susceptibility locus [7].

Immunophenotyping in *Atxn1*-null EAE mice highlighted the significant expansion of the B-1a population. This particular B cell subset is characterized by CD5 and CD11b expression, secretes most of the circulating natural IgM antibodies [8], and its dysregulation has been implicated in several autoimmune diseases, including MS [9]. B-1a cells also modulate the severity of the EAE phenotype [10]. We report here the transcriptional activity of B-1a cells in wild-type and *Atxn1*-null mice following an encephalitogenic challenge.

## Results

**Fig. 1:**
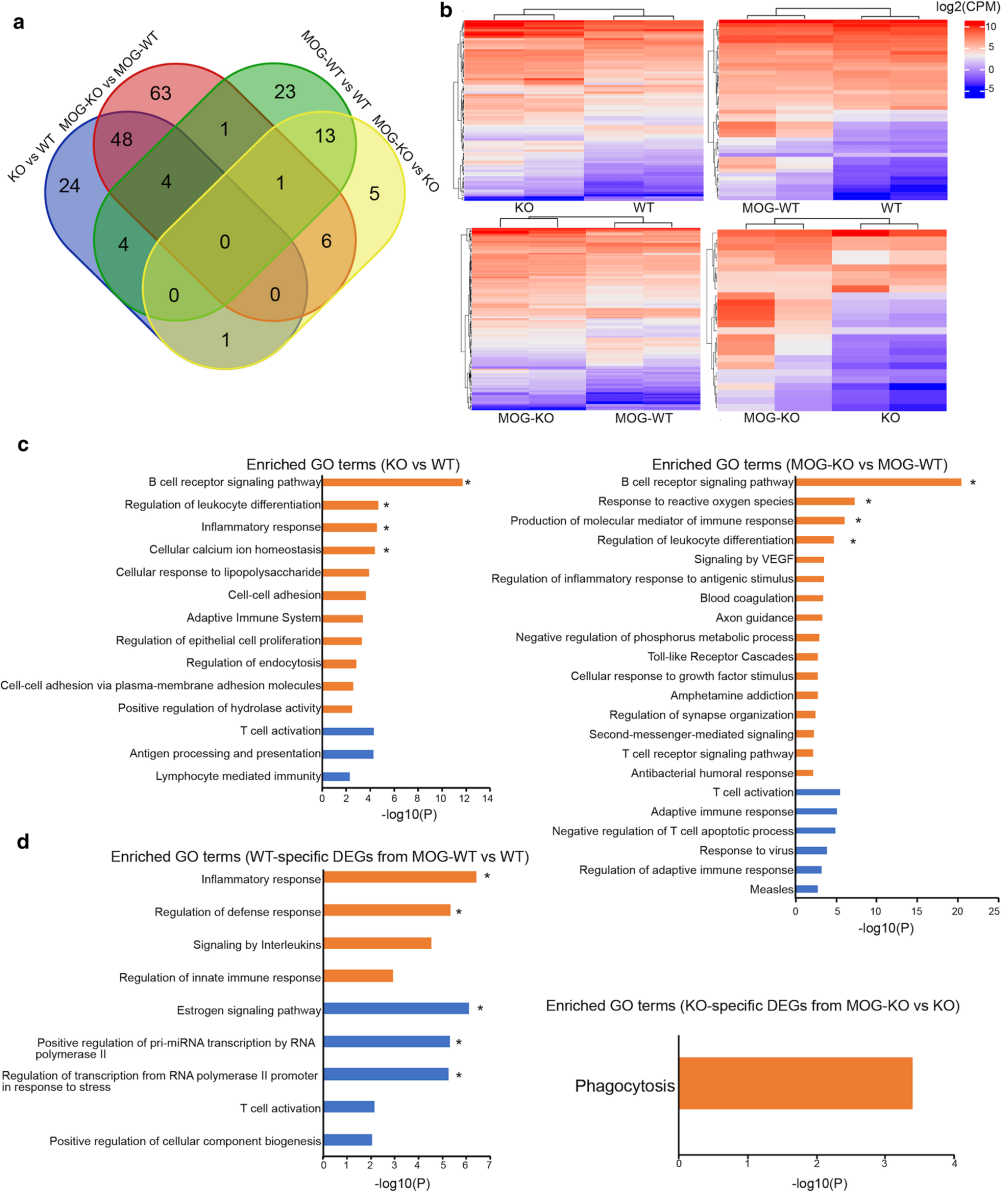
Ataxin-1 controls immunoglobulin transcription and antigen presentation in B-1a cells. **a** Venn diagrams showing the overlap of DEGs between *Atxn1*-null and wildtype B-1a cells in both cross-sectional and longitudinal comparisons. **b** Unsupervised clustering of the DEGs separates *Atxn1*-null and wildtype B-1a cells at baseline and 10 days post-immunization (dpi) with MOG peptide (heatmaps on the left). Clustering also separates B-1a cells between baseline and post-immunization conditions within each genotype (heatmaps on the right). **c** Histograms showing the significant GO terms associated with cross-sectional comparisons at baseline and 10 dpi. **d** Histograms showing the significant GO terms associated with the *Atxn1*-null and wildtype specific DEGs from longitudinal comparisons. Significant GO terms enriched in up-regulated genes are depicted in orange, while the GO terms enriched in down-regulated genes are depicted in blue. Asterisks indicate the GO terms that passed multiple comparison correction (Q values less than 0.05)

B-1a cell isolation and transcriptome profiling were carried out as detailed in Additional file 1 and Additional file 2. In the cross-sectional comparison between wildtype and knockout B-1a cells, 81 differentially expressed genes (DEGs) were identified at baseline (62 up- and 19 down-regulated), while 123 DEGs were detected upon MOG peptide immunization (84 up- and 39 down-regulated) (Additional file 3), with an overlap of 52 genes (Fig. 1a). These differences are sufficient to clearly separate the two genotypes by unsupervised clustering, with 100% approximately unbiased probability in both conditions (Fig. 1b). Similar results were obtained using the full transcriptomes (Additional file 4). Gene ontology (GO) enrichment for the up-regulated genes in *Atxn1*-null B-1a cells highlighted “B cell receptor signaling pathway” as the most significant category at baseline and upon MOG peptide-stimulation (Fig. 1c), principally due to the upregulation of immunoglobulin genes (Additional file 5). No category survived multiple correction in the GO analysis on the down-regulated genes in both conditions (Fig. 1c).

A suggestive association (uncorrected P < 0.001) with up-regulated genes in the VEGF signaling pathway was identified in MOG peptide-stimulated B-1a cells (Fig. 1c and Additional file 5). This pathway is crucial for cell survival and its chronic activation is typically associated with malignant CD5^+^ B cell accumulation in chronic lymphocytic leukemia (CLL) [11]. Thus, aberrant VEGF signaling may also contribute to B-1a expansion in *Atxn1*-null mice in the context of adaptive immune response towards CNS antigens.

To capture those genes dynamically regulated upon the immune response, we then compared the transcriptomic profiles at baseline and upon MOG peptide immunization within each genotype. In this second analysis, 46 DEGs were found in wildtype B-1a cells (28 up- and 18 down-regulated) and 26 DEGs in knockout B-1a cells (21 up- and 5 down regulated) (Additional file 3), with an overlap of 14 genes (Fig. 1a). Also, in this case the differences produced a clear clustering (Fig. 1b and Additional file 4). GO analysis on the wildtype-specific DEGs found “inflammatory response” and “estrogen signaling pathway” as the most significantly enriched categories for the up- and down-regulated genes, respectively (Fig. 1d and Additional file 5). A nominally significant association with the “phagocytosis” category was found for knockout-specific up-regulated genes (Fig. 1d and Additional file 5).

At the molecular level, ataxin-1 works in concert with the transcriptional repressor capicua (CIC) to inhibit the expression of target genes. Consequently, we screened the promoter regions of all the DEGs for the presence of two known CIC-binding motifs, TGAATGAA and TGAATGGA, and observed a significant enrichment for the second target sequence upon MOG peptide immunization (Additional file 6). These results indicate that DEGs in activated B-1a cells likely represent ataxin-1 direct targets while the genes found dysregulated at baseline are downstream effectors. This hypothesis is further supported by the evidence that a motif enrichment was found in longitudinally down-regulated genes from wildtype but not knockout B-1a cells (Additional file 6). Interestingly, our previous analysis performed in the whole B cell population showed an enrichment for CIC-binding motifs only at baseline [6].

## Conclusion

Here, we extended our characterization of ataxin-1 immunomodulatory functions by reconstructing the genetic programs controlled by this protein in B-1a cells, a particular B cell subset that is pathologically expanded in *Atxn1*-null mice. We show that the immunoglobulin transcriptional machine in B-1a cells is under the transcriptional control of ataxin-1, either in basal conditions or upon an encephalitogenic challenge. This is in agreement with the elevated IgG and IgM serum titers that we have previously measured in *Atxn1*^*−/−*^ mice [6]. Notably, intrathecal IgM production is a strong risk factor for clinically isolated syndrome (CIS) to MS conversion [12]. Hence, ataxin-1 expression in B-1a cells might affect disease risk by modulating IgM levels.

The longitudinal gene expression analysis highlights the putative role of ataxin-1 in controlling the phagocytic activity of B-1a cells. In addition to immunoglobulin secretion, B-1a cells have been demonstrated to effectively internalize and present antigens to CD4^+^ T cells [13]. Therefore, ataxin-1 may also regulate the antigen presenting cell (APC) function of B-1a cells in a pro-inflammatory milieu.

Lastly, we observed the dysregulation of VEGF signaling in *Atxn1*-null B-1a cells. VEGF was shown to exert opposite effects in MS and EAE, either detrimental or protective, depending on the specific isoforms and receptors involved [14]. Intriguingly, this cascade was first found dysregulated in Purkinje neurons of a SCA1 knock-in model [15], pinpointing possible mechanistic intersects between neurodegenerative and autoimmune processes.

## Supplementary Information

**Additional file 1.** Materials and methods.

**Additional file 2.** Expression levels for genes specific of B-1a cells (*Cd19*, *Cd43* and *Cd5*), T cells (*Cd4*, *Cd8a* and *Cd8b1*) and monocytes (*Cd11b*). The levels are expressed as count per million reads (CPM) and represent mean values ± SD across all the datasets (N=8).

**Additional file 3.** Significant differentially expressed genes (DEGs) in *Atxn1*-null and wildtype B-1a cells.

**Additional file 4.** **a** Unsupervised clustering of full transcriptomes separates *Atxn1*-null and wildtype B-1a cells at baseline and 10 days post-immunization (dpi) with MOG peptide. Clustering also separates B-1a cells between baseline and post-immunization conditions within each genotype. **b** Overlap between the DEGs identified with the edgeR and DESeq2 packages.

**Additional file 5.** List of significant gene ontology (GO) terms from cross-sectional and longitudinal comparisons.

**Additional file 6.** Enrichment analysis for CIC binding-motifs in the promoters of DEGs from cross-sectional and longitudinal comparisons.

## Data Availability

The datasets used and/or analyzed during the current study are available from the corresponding author on reasonable request.

## Abbreviations

AD: Alzheimer’s disease
APC: Antigen presenting cell
BCR: B cell receptor
CD4: Cluster of differentiation 4
CIC: Capicua
CIS: Clinically isolated syndrome
CLL: Chronic lymphocytic leukemia
CNS: Central nervous system
DEGs: Differentially expressed genes
Dpi: Day post-immunization
EAE: Experimental autoimmune encephalomyelitis
ERK: Extracellular signal-regulated kinase
GO: Gene ontology
MOG: Myelin oligodendrocyte glycoprotein
MS: Multiple sclerosis
SCA1: Spinocerebellar ataxia type 1
STAT: Signal transducer and activator of transcription
VEGF: Vascular endothelial growth factor
