# Supplementary material for: The novel multiple sclerosis susceptibility gene ATXN1 regulates B cell receptor signaling in B-1a cells
Source: Mol Brain. 2021 Jan 21;14:19. doi: 10.1186/s13041-020-00715-0 (PMC7819313; doi:10.1186/s13041-020-00715-0)
Supplement: Supplementary file 1 — Additional file 1. Materials and methods. [file 13041_2020_715_MOESM1_ESM.docx]

**Additional file**

**The novel multiple sclerosis susceptibility gene *ATXN1* regulates B cell receptor signaling in B-1a cells**

Qin Ma^1^, Alessandro Didonna^1^*

^1^Weill Institute for Neurosciences, Department of Neurology, University of California, San Francisco, CA 94158, USA

***Corresponding Author:** Alessandro Didonna, Department of Neurology and Weill Institute for Neurosciences, University of California San Francisco, 675 Nelson Rising Lane, San Francisco, CA 94158, USA. Phone: +1-415-502-7211; Email: alessandro.didonna@ucsf.edu

**This file includes:**

**Materials and Methods**

**Other additional file for this manuscript includes:**

**Additional file 2.** Expression levels for genes specific of B-1a cells (*Cd19*, *Cd43* and *Cd5*), T cells (*Cd4*, *Cd8a* and *Cd8b1*) and monocytes (*Cd11b*). The levels are expressed as count per million reads (CPM) and represent mean values ± SD across all the datasets (N=8).

**Additional file 3.** Significant differentially expressed genes (DEGs) in *Atxn1*-null and wildtype B-1a cells.

**Additional file 4. a** Unsupervised clustering of full transcriptomes separates *Atxn1*-null and wildtype B-1a cells at baseline and 10 days post-immunization (dpi) with MOG peptide. Clustering also separates B-1a cells between baseline and post-immunization conditions within each genotype. **b** Overlap between the DEGs identified with the edgeR and DESeq2 packages.

**Additional file 5.** List of significant gene ontology (GO) terms from cross-sectional and longitudinal comparisons.

**Additional file 6.** Enrichment analysis for CIC binding-motifs in the promoters of DEGs from cross-sectional and longitudinal comparisons.

**Materials and Methods**

**Mouse strains**

*Atxn1^-/-^* mice (B6.129S7-*Atxn1^tm2Hzo^*/J) were a kind gift of Dr. Huda Zoghbi (Baylor College of Medicine, Houston, TX) and were previously described (1). To avoid possible confounding affects, mice were kept on a pure C57BL/6J background and only females were used for experiments. Mice were housed in a specific pathogen free (SPF) facility and all procedures were performed in compliance with experimental guidelines approved by the University of California San Francisco Committee on Animal Research (CAR).

**RNA-seq**

B-1a cells were purified by immunomagnetic selection using the B-1a Cell Isolation Kit (Miltenyi) from the spleens of *Atxn1^-/-^* and wildtype mice of 8-10 weeks of age, either naïve or after 10 days from immunization with 100 μg of MOG_35-55_ peptide in complete Freund’s adjuvant (CFA). Pools of 3 spleens were used in each isolation and two biological replicates were generated for each genotype and condition. The purity of isolated B-1a cells was further confirmed by analyzing the normalized expression levels for genes specific of B-1a (*Cd19*, *Cd43,* and *Cd5*), T cells (*Cd4*, *Cd8a*, and *Cd8b1*) and monocytes (*Cd11b*) (Additional file 2). Total RNA was extracted with the RNeasy Micro Kit (Qiagen) and next-generation sequencing of the poly(A) fractions was performed on a BGISEQ platform (BGI Genomics), generating 40 million of pair-end 100 bp reads per sample. After trimming low-quality and adaptor sequences with the Trimmomatic tool, the remaining reads were aligned to the mouse genome (mm10) using hisat2-2.1.0. The htseq-count tool and Ensembl gtf file were used to count aligned reads for each gene. Differentially expressed genes (DEGs) were identified using the edgeR package. P values less than 0.05 after false discovery rate (FDR) correction were considered significant. The results were independently validated using the DESeq2 package, which found an overlap in DEGs between 63% and 89% among the different comparisons (Additional file 4).

**Bioinformatic analyses**

Pathway analysis was carried out using Metascape, a web-based portal providing comprehensive gene annotation and enrichment analysis including GO processes, KEGG pathways, and the Reactome gene set (2). To check for enrichment in CIC binding sites, the promoter regions (0.5 kb upstream the TSS) of all DEGs were extracted from the mouse genome assembly mm10 and then used as inputs for the FIMO software (http://meme-suite.org/tools/fimo), looking for exact matches with the CIC consensus motif TGAATG(A/G)A on both DNA strands. Statistical significance was tested by calculating the frequency distribution of CIC motifs identified across 1000 random samples of expressed protein-coding genes of the same size of each DEG list.

**References**

1. Matilla A, Roberson ED, Banfi S, Morales J, Armstrong DL, Burright EN, et al. Mice lacking ataxin-1 display learning deficits and decreased hippocampal paired-pulse facilitation. J Neurosci. 1998;18(14):5508-16.

2. Zhou Y, Zhou B, Pache L, Chang M, Khodabakhshi AH, Tanaseichuk O, et al. Metascape provides a biologist-oriented resource for the analysis of systems-level datasets. Nat Commun. 2019;10(1):1523.
