# Supplementary figures and images for: The novel multiple sclerosis susceptibility gene ATXN1 regulates B cell receptor signaling in B-1a cells
Source: Mol Brain. 2021 Jan 21;14:19. doi: 10.1186/s13041-020-00715-0 (PMC7819313; doi:10.1186/s13041-020-00715-0)

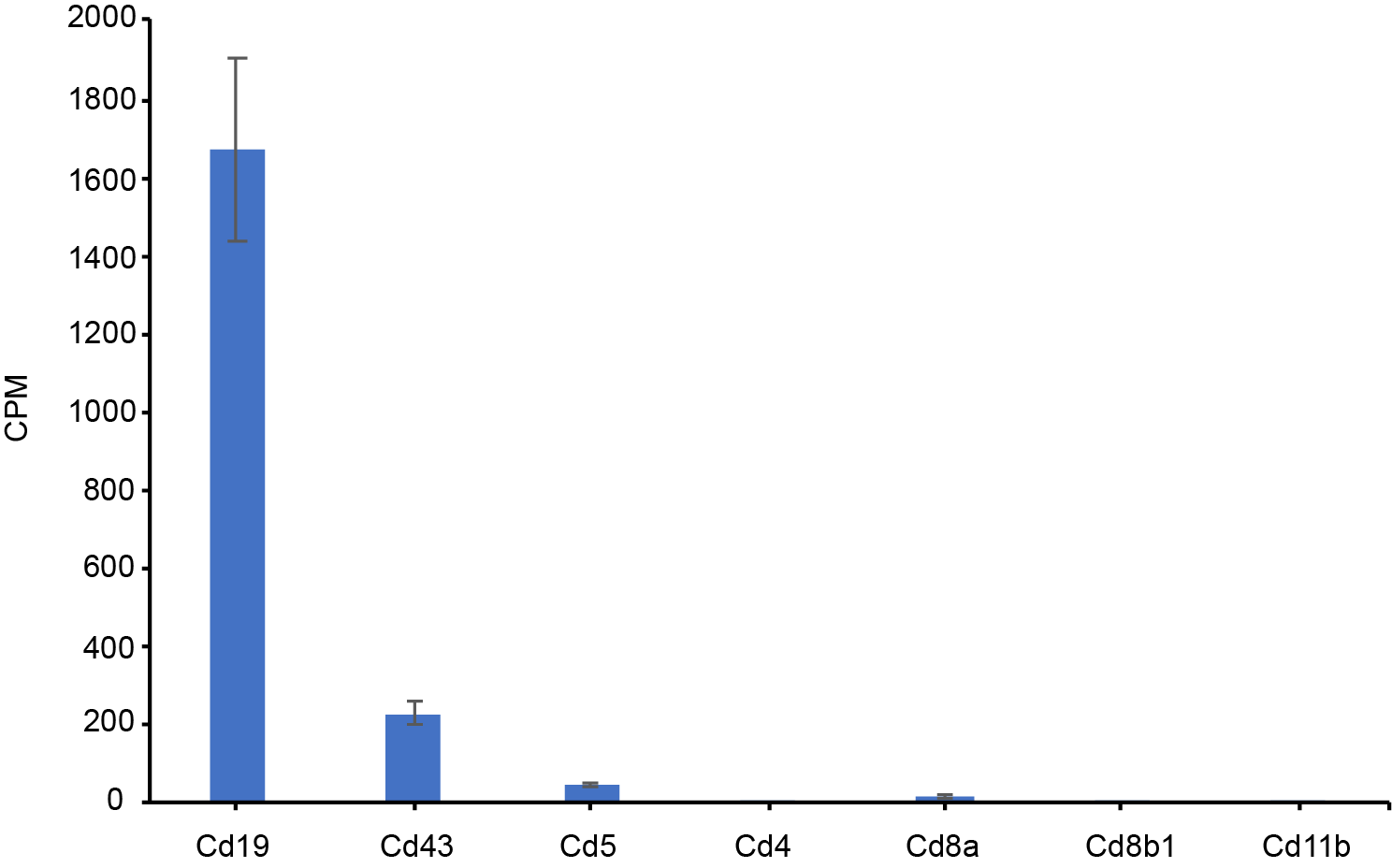

Supplement: Supplementary file 2 — Additional file 2. Expression levels for genes specific of B-1a cells (Cd19, Cd43 and Cd5), T cells (Cd4, Cd8a and Cd8b1) and monocytes (Cd11b). The levels are expressed as count per million reads (CPM) and represent mean values ± SD across all the datasets (N=8). [file 13041_2020_715_MOESM2_ESM.png]

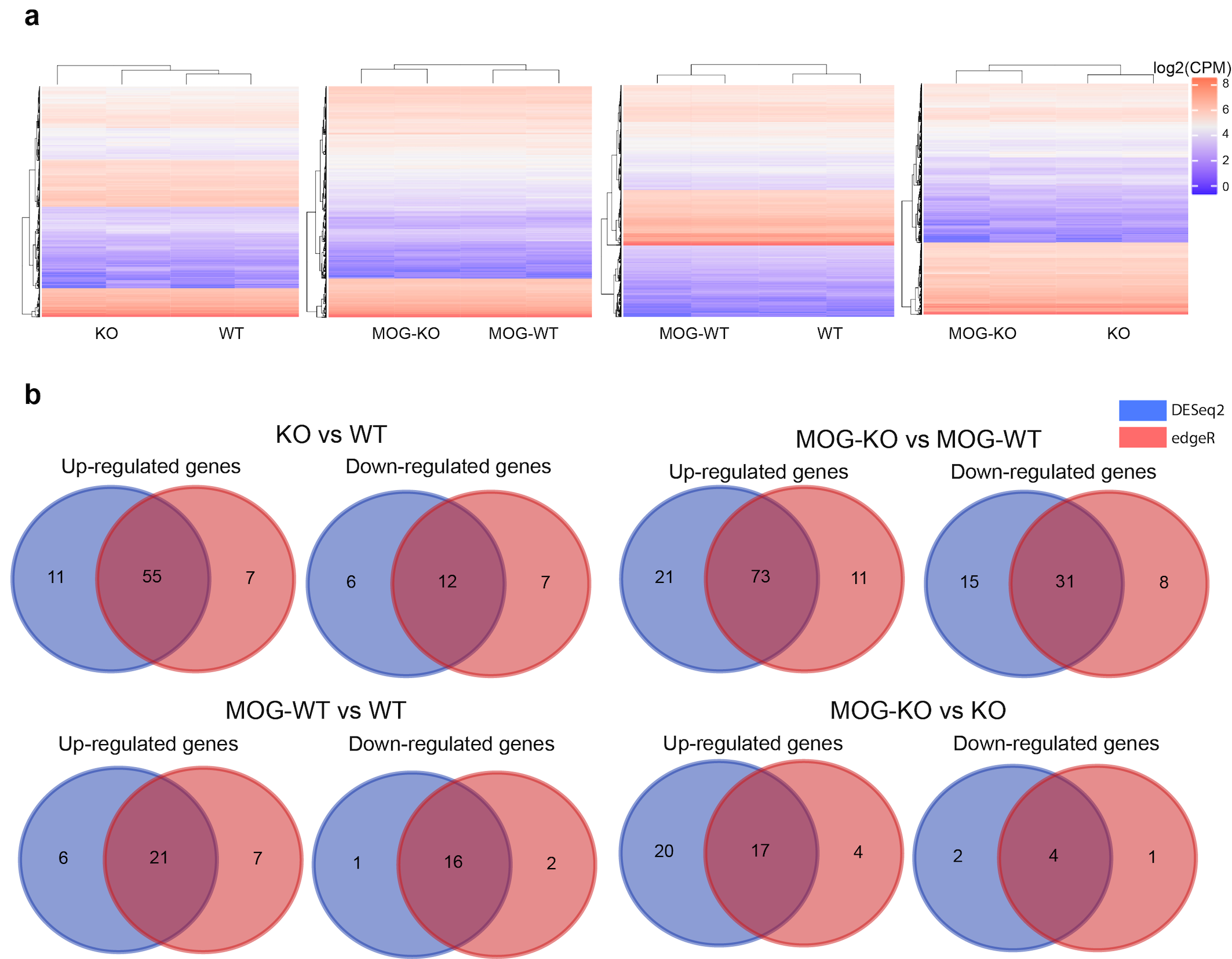

Supplement: Supplementary file 4 — Additional file 4. a Unsupervised clustering of full transcriptomes separates Atxn1-null and wildtype B-1a cells at baseline and 10 days post-immunization (dpi) with MOG peptide. Clustering also separates B-1a cells between baseline and post-immunization conditions within each genotype. b Overlap between the DEGs identified with the edgeR and DESeq2 packages. [file 13041_2020_715_MOESM4_ESM.png]
